# Development and Validation of a Gas Chromatography Method for the Trace Level Determination of Allylamine in Sevelamer Hydrochloride and Sevelamer Carbonate Drug Substances

**DOI:** 10.3797/scipharm.1309-12

**Published:** 2013-11-14

**Authors:** Raju V. S. N. Kadiyala, Pavan Kumar S. R. Kothapalli, Madhava Reddy Peddolla, Pradeep Rajput, Hemant Kumar Sharma, Shankar Reddy Budeti, Himabindu Gandham, Annapurna Nowduri

**Affiliations:** 1 Aurobindo Pharma Ltd Research Centre-II, Survey No: 71 & 72, Indrakaran village, Sangareddy mandal, Medak district, 502329, Andhra Pradesh, India.; 2 Andhra University College of Engineering, Visakhapatnam-530003, Andhra Pradesh, India.

**Keywords:** Allylamine, Sevelamer hydrochloride, Sevelamer carbonate, Gas chromatography with Flame ionization detector

## Abstract

A capillary gas chromatography method using a flame ionization detector has been developed for the trace analysis of allylamine (AA) in sevelamer hydrochloride (SVH) and sevelamer carbonate (SVC) drug substances. The method utilized a mega bore capillary column DB-CAM (30 m × 0.53 mm × 1.0 μm) with a bonded and cross-linked, base-deactivated polyethylene glycol stationary phase and was validated for specificity, sensitivity, precision, linearity, and accuracy. The detection and quantitation limits obtained for allylamine were 2 μg/g and 6 μg/g, respectively. The method was found to be linear in the range between 6 μg/g and 148 μg/g with a correlation coefficient of 0.9990. The average recoveries obtained in SVH and SVC were 93.9% and 99.1%, respectively. The developed method was found to be robust for the determination of AA in sevelamer drug substances and also the specificity was demonstrated with a gas chromatograph coupled with a mass spectrometer.

## Introduction

**Fig. 1:**
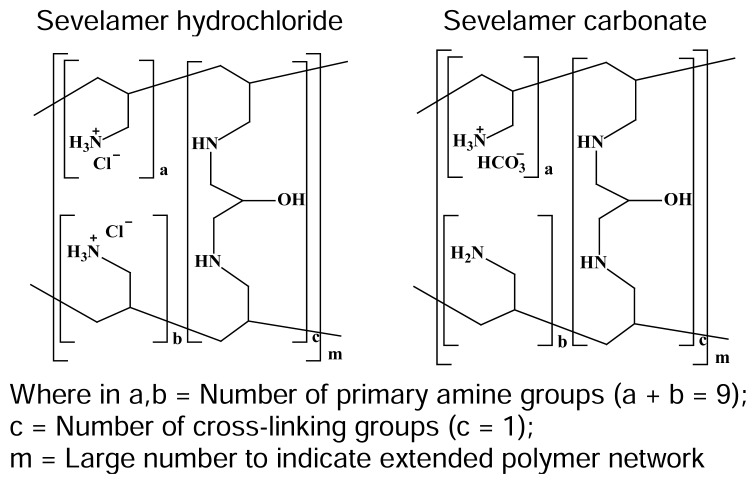
Chemical structures of sevelamer hydrochloride and sevelamer carbonate. Where in a,b = Number of primary amine groups (a + b = 9); c = Number of cross-linking groups (c = 1); m = Large number to indicate extended polymer network

Sevelamer is known as poly(allylamine) cross-linked with epichlorohydrin. Sevelamer is available in the form of hydrochloride salt as well as carbonate salt (sevelamer hydrochloride and sevelamer carbonate) and both are insoluble in water [1]. Sevelamer hydrochloride (SVH) was marketed as Renager® and sevelamer carbonate (SVC) was marketed as Renvela® respectably by the Genzyme Corporation. These are orally active drug substances which are used for the treatment of hyperphosphatemia. Sevelamer acts as a phosphate binder and it has been shown to decrease serum phosphate concentrations in patients with chronic kidney disease [2]. The chemical structures of sevelamer hydrochloride and sevelamer carbonate are shown in Figure 1.

Poly(allylamine hydrochloride) is an intermediate in the synthetic process of SVH and SVC drug substances. In the preparation of poly(allylamine hydrochloride), allylamine (AA) is used as a key starting material, which is an unsaturated aliphatic amine, a colorless liquid, and chemically known as 3-amino-1-propene. It is potentially toxic and its oral LD_50_ is 106 mg/kg for rats. The general toxic effects of the freebase forms are primarily related to irritation of the mucous membranes, whereas the relatively long history of experimental use of this chemical has emphasized its extraordinarily deleterious effects on the heart and vascular tissue [3]. Its acute toxicity is believed to be involved in the metabolism of AA to highly reactive acrolein. Because of its known toxic nature, the presence of residual AA in SVH and SVC drug substances should be controlled as per ICH (International Conference on Harmonization), Food and Drug Administration (FDA), and European Medicines Agency Guidelines (EMAG) [4–6].

Some of the analytical methods are already available in literature for the determination of AA and aliphatic amines. Recently, the United States Pharmacopeial forum has published a HPLC (High Pressure Liquid Chromatography) derivatization method (with sodium tetraborate solution) using a fluorescence detector [7] for the quantification of allylamine. An ion chromatography method with a conductivity detector was published for trace level determination of allylamine with the limit of detection and limit of quantitation values 2.7 and 9.0 μg/ml, respectively [8]. Kurt Andersson has reported the determination of a trace amount of n-propylamine, n-butylamine, and allylamine in air samples by an HPLC reversed-phase method with UV (Ultraviolet-visible) detection using 1-naphthyl isocyanate derivatives [9]. The determination of aliphatic amines in water by gas chromatography using headspace solvent microextraction was reported by Massoud Kaykhaii [10]. The quantification of residual aliphatic amines in oligonucleotides was reported on headspace gas chromatography (GC) [11]. The determination of short aliphatic amines in water by HPLC with chemiluminescence detection [12] was reported by S. Meseguer Lloret. Although different methods were reported in the literature for the determination of AA in different samples, this research paper describes a simple and sensitive GC method with a flame ionization detector (FID) for the quantification of AA in SVH and SVC drug substances with limit of detection and limit of quantitation values 0.3 and 1.0 μg/ml, respectively, which is more sensitive when compared with the ion chromatography (IC) method [8]. Considering a maximum daily dosage of 7.2 g per day of sevelamer drugs, any impurity in sevelamer drugs must be less than 0.05%, but 100 μg/g has been chosen as the specification level for this research work. The developed method was validated for specificity, sensitivity (limit of detection and limit of quantitation), linearity, precision (system precision, method precision, and intermediate precision), accuracy, and robustness in accordance with ICH Q2 (R1) / United States Pharmacopeia (USP) guidelines [13, 14].

## Experimental

### Chemicals and Reagents

The SVH and SVC drug substances were gifted by the Aurobindo Pharma Limited Research Centre (Hyderabad, India). Triethylamine and allylamine were purchased from Sigma-Aldrich (Steinheim, Germany). Analytical grade methylene chloride, chloroform, sodium carbonate, sodium hydroxide pellets, HPLC grade dimethyl sulfoxide, and water were procured from Merck chemicals (Mumbai, India). Sodium sulfate anhydrous was purchased from RFCL limited (New Delhi, India).

### Instrumentation for GC-FID

The Agilent 7890A equipped with a flame ionization detector and a 7683B auto sampler was used in the research work. Data acquisition and processing were conducted using the EZ chrom software.

### Instrumentation for GC-MS

The Shimadzu GC coupled with a GCMS-QP 2010 plus mass detector (GC-MS) and a Combipal auto sampler was used in the research work. Data acquisition and processing were conducted using GCMS solution software.

### Operating Conditions for GC-FID

The GC separation was carried out on a J&W Scientific DB-CAM column with dimensions of 30 m length, 0.53 mm I.D., and film thickness 1.0 μm with an injection volume of 2 μL. The oven temperature gradient was started at 70°C and held for 6 min. Then it was raised to 200°C at the rate of 20°C/min and held at 200°C for 7.5 min. Helium was used as a carrier gas with a constant flow rate of 5.33 ml/min with split mode 1:5. The injector temperature and the detector temperature were kept at 220°C and at 260°C, respectively.

### Operating Conditions for GC-MS

The J&W Scientific DB-CAM capillary column with a dimension of 30 m × 0.25 mm × 0.25 μm film thickness was used for the chromatographic separation. The initial oven temperature was 60°C, maintained for 6 min, and then increased to 200°C at a rate of 10°C/min followed by holding at 200°C for 1 min. The injection volume was 2 μl with split mode 1:5. Helium was used as the carrier gas with a constant flow rate of 0.49 ml/min. The injector temperature was 200°C. The ion source and interface temperatures were set at 250 and 220°C, respectively. The electron impact (EI) mode at 70 eV was utilized for sample ionization. The GC-MS spectra for AA and internal standard were obtained through the injection of the standard and sample solutions and scanning in the range of m/z 30–700.

### Preparation of Solutions

#### 2 N Sodium Hydroxide Solution

Accurately weigh and transfer 8.0 g of sodium hydroxide pellets into a 100 ml volumetric flask containing about 50 ml of water, dissolve, and then dilute to volume with water.

#### Internal Standard Solution

Accurately weigh and transfer 40 mg of triethylamine into a 25 ml volumetric flask containing about 10 ml of chloroform, then dilute to volume with chloroform. Further dilute 1.0 ml of this solution to 200 ml with chloroform (8 mg/ml).

#### Preparation of Blank Solution

Transfer 3 ml of internal standard solution into a 10 ml centrifuge tube and add 4 ml of 2 N sodium hydroxide solution and centrifuge for 5 min at 3000 rpm. Collect the lower layer (chloroform layer) carefully through an automatic pipette and pass through anhydrous sodium sulfate.

#### Preparation of Standard Solution (17 mg/ml)

Accurately weigh and transfer 42 mg of allylamine into a 50 ml volumetric flask containing about 20 ml of internal standard solution, then dilute to volume with internal standard solution. Further dilute 1.0 ml of this solution to 50 ml with internal standard solution.

Transfer 3 ml of standard solution into a 10 ml centrifuge tube and add 4 ml of 2 N sodium hydroxide solution and centrifuge for 5 min at 3000 rpm. Collect the lower layer (chloroform layer) carefully through an automatic pipette and pass through anhydrous sodium sulfate.

#### Preparation of Sample Solution

Accurately weigh and transfer 500 mg of the sample into a 10 ml centrifuge tube, add 4 ml of 2 N sodium hydroxide solution. Shake the contents for approximately 5 min mechanically. Transfer 3.0 ml of the internal standard solution into the centrifuge tube. Again, shake the contents for approximately 5 min mechanically and centrifuge for 5 min at 3000 rpm. Collect the lower layer (chloroform layer) carefully through an automatic pipette and pass through anhydrous sodium sulfate.

## Results and Discussions

### Method Development and Optimization

The challenge is to achieve the detection and quantitation at a low level using the gas chromatograph with a flame ionization detector (GC-FID) for obtaining good separation and the desired sensitivity. Development trials were initiated on the head-space technique using the stationary phase, 5% diphenyl, 95% dimethyl polysiloxane (Rtx-5; Make: Restek) as this column is an amine column specific for amines and other basic compounds. Standard AA solution and spiked sample solutions were prepared and injected into the GC. AA was eluted at around 5 min and unknown peak interference was observed at AA retention time in the spiked sample chromatogram. Further different trials were performed by changing the temperature programmes, column dimensions, and stationary phases. However, interference was observed. Later on, the direct-liquid injection technique was selected for the quantification of AA. The sample solution was prepared by dissolving the sample in chloroform, filtering, and injecting into the GC. Background interference was encountered in this trial. After cleaning the inlet port (to avoid ghost peaks), a broad peak shape of AA was observed, which suggests another type of sample preparation to reduce the interference from the sample matrix for quantification and proper peak shape purposes. The extraction technique using internal standard has been chosen for the low level quantification of AA by the liquid injection technique. Triethylamine (TEA) was chosen as the internal standard as it does not react with AA and it has a similar functional group. Initially, methylene chloride was chosen as an extraction solvent, but some interference was observed at the retention time of the internal standard in the DB-CAM (30 m × 0.53 mm × 1.0 μm) column. When chloroform was used as an extraction solvent, there was no interference found at the retention time of TEA and AA. The resolution between TEA and AA peaks should not be less than 2.0, which is kept as the system suitability criteria. Finally, the DB-CAM (30 m × 0.53 mm × 1.0 μm) (Make: J&W, fused silica coated with base-deactivated polyethylene glycol) column was preferred, as the analyte peaks were eluted with shorter retention times when compared with smaller internal diameter columns.

### Method Validation

The developed and optimized method was validated for specificity, sensitivity [limit of detection (LOD) & limit of quantitation (LOQ)], linearity, precision [system precision, method precision & intermediate precision], accuracy, and robustness as per ICH guideline Q2(R1) [13] and USP<1225> [14].

### Specificity

**Fig. 2:**
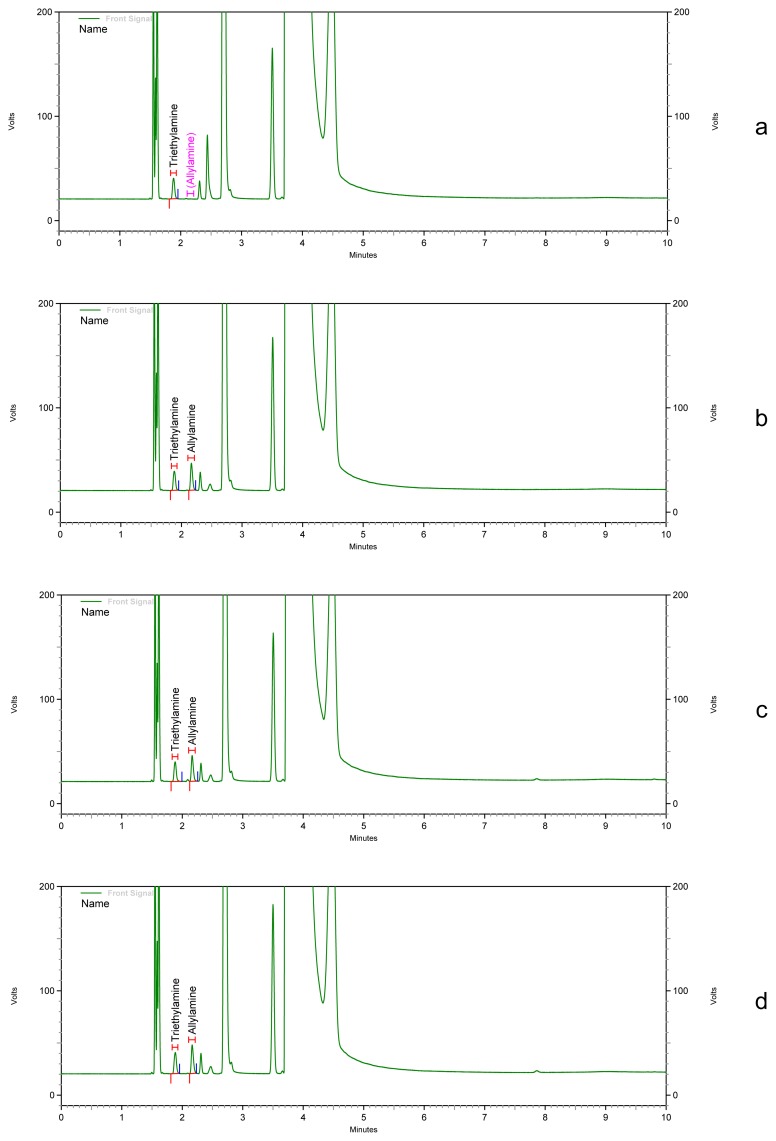
Typical GC chromatograms of a) blank solution, b) standard solution, c) sevelamer hydrochloride spiked with allylamine including known residual solvents, and d) sevelamer carbonate spiked with allylamine including known residual solvents.

**Fig. 3:**
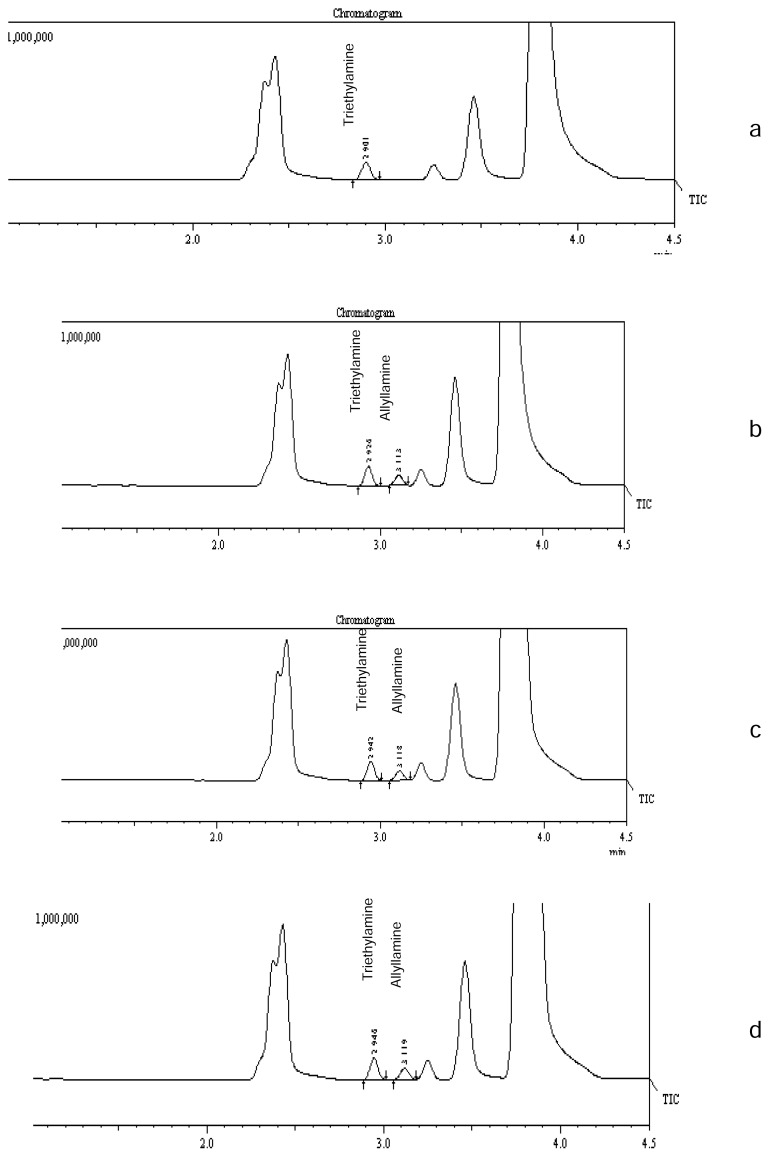
Typical GC-MS total ion chromatograms of a) blank solution, b) standard solution, c) sevelamer hydrochloride spiked with allylamine, and d) sevelamer carbonate spiked with allylamine.

The blank solution, individual injections of AA, and all other known residual solvents (which are used in the process of sevelamer drugs i.e. methanol and epichlorohydrin), SVH and SVC drug substance sample solutions, control sample solutions (SVH and SVC drug substances spiked with AA), and spiked sample solutions (SVH and SVC drug substances spiked with AA and all other known residual solvents) were prepared and injected into the GC. From the chromatograms, it was found that the AA peak was well-separated from all other known solvents, indicating that the test method is selective and specific for the determination of AA in sevelamer compounds. Typical GC chromatograms of the blank solution, standard solution, and spiked sample solutions of SVH and SVC are shown in Figure 2. The identity and specificity of the AA peak was further demonstrated through GC-MS. The internal standard solution, mass fragments including the base peak of the AA standard and sevelamer compounds spiked with AA were found to be comparable with National Institute of Standards Technology (NIST) mass spectral library for AA. Typical GC-MS ion chromatograms of the blank solution, standard solution, and sevelamer compounds spiked with AA are shown in Figure 3. From the GC and GC-MS data, it is concluded that the proposed method is specific for allylamine determination.

### LOD and LOQ Precision

The limit of detection (LOD) and limit of quantification (LOQ) values for AA were determined by the signal-to-noise ratio (s/n) method. The minimum concentration at 3:1 s/n was considered as the LOD and the concentration at 10:1 s/n was considered as the LOQ. The LOD and LOQ values obtained for AA were 2 and 6 μg/g, respectively, with respect to the sample concentration, which corresponds to 0.3 and 1.0 μg/ml. Precision was verified by preparing the solutions at about the LOD and LOQ concentrations and injecting each solution six times into the GC. The results are tabulated in Table-1.

### Linearity

The linearity was evaluated by measuring the area ratio for AA to internal standard over the range of 1.0 to 25 μg/ml [6 to 150 μg/g with respect to sample concentration] and the obtained data were subjected to statistical analysis using a linear regression model. The statistical results such as correlation coefficient, slope, intercept, STEYX are reported in Table 1.

### Accuracy

Accuracy of the method was verified through performing recovery experiments by spiking known amounts of AA at the LOQ level, 50%, 100%, and 150% of the specification level (i.e. 100 μg/g) to SVH and SVC drugs separately and the obtained recovery results are tabulated in Table 2, respectively.

### Precision

The system precision was demonstrated by injecting the standard solution of AA six times into GC and calculating the area ratios using the areas obtained from AA and TEA. The method precision of the method was established by preparing six individual sample preparations by spiking AA to SVH and SVC drug substances separately, injecting into the GC, and calculating the AA content. The ruggedness of the method was evaluated by preparing six individual sample preparations [the same samples which were used in the method precision experiment] by spiking AA to SVH and SVC drug substances separately, injecting into the GC, and calculating the AA content using two different columns, different makes of instruments (i.e. Agilent and Shimadzu), and by different analysts on different days. The achieved precision experimental results are reported in Table 3.

### Robustness

This study was performed by making deliberate variations in the method parameters. The effect of variation in the carrier gas flow and column initial oven temperature for the AA determination was studied. All experimental system suitability results (resolution between TEA and AA) are mentioned in Table 4.

## Conclusion

The method validation data demonstrated that the developed GC method is more sensitive than the reported ion chromatography method (8). Also, the specificity of the method was established by both GC and GC-MS as well as accurate for the estimation of AA. Hence, the validated GC method can be employed in the routine analysis for the quantification of allylamine in sevelamer hydrochloride and sevelamer carbonate drug substances.

## Figures and Tables

**Tab. 1:** Statistical data of linearity, LOD/LOQ for allylamine

Statistical parameters	Results
Correlation coefficient	0.9990
Concentration range (μg/g)	6–148
Intercept	0.0231
Slope(S)	0.0125
Residual standard on deviation response (SD)	0.0322
Limit of detection (μg/g)	2
Limit of quantification (μg/g)	6
Precision for Limit Of Detection (%R.S.D)	3.1
Precision for Limit Of Quantification (%R.S.D)	2.6
Calibration points	6

**Tab. 2:** Accuracy data of allylamine in sevelamer hydrochloride and sevelamer carbonate

Accuracy (Average of 3 replicates)	Sevelamer hydrochloride			

	Level-I	Level-II	Level-III	Level-IV
Added (μg/g )	6.1	50.5	100.9	151.6
Recovered (μg/g )	5.8	47.5	94.8	140.6
Recovery (%)	95.1	94.1	94.0	92.7
R.S.D(%)	2.0	0.0	0.3	0.1
Overall recovery (%) (Average of 12 replicates)	93.9			

**Accuracy (Average of 3 replicates)**	Sevelamer carbonate			

	**Level-I**	**Level-II**	**Level-III**	**Level-IV**

Added (μg/g )	6.1	50.4	101.1	151.5
Recovered (μg/g )	6.0	50.1	98.3	153.8
Recovery (%)	98.4	99.4	97.2	101.5
R.S.D(%)	1.7	0.4	0.2	0.8
Overall recovery (%) (Average of 12 replicates)	99.1			

**Tab. 3:** Statistical data of precision experiments

		Sevelamer hydrochloride		Sevelamer carbonate	

ID	System precision^a^	Method precision^b^	Ruggedness^b^	Method precision^b^	Ruggedness^b^

		(μg/g)		(μg/g)	
1	1.2471	94	99	97	100
2	1.2390	95	100	97	100
3	1.2368	95	99	97	100
4	1.2352	95	101	99	99
5	1.2332	95	97	99	98
6	1.2324	95	99	100	97
Mean	1.2373	95	99	98	99
SD	0.0054	0.4	1.3	1.3	1.3
% RSD	0.4	0.4	1.3	1.3	1.3
95% CI(±)	0.0057	0.4	1.4	1.4	1.4

Overall statistical data (n=12)	Mean	97		99	
	SD	2.4		1.3	
	% RSD	2.5		1.3	
	95% CI(±)	1.5		0.8	

a area ratio of allylamine;

b content of allylamine.

**Tab. 4:** Summary of system suitability results

Experiment	Resolution between Triethylamine and Allylamine
1^st^ day	4.4
2^nd^ day	4.9
3^rd^ day	4.5
4^th^ day	4.6
−10% of carrier gas flow	5.3
+10% of carrier gas flow	4.5
−2°C column initial oven temperature	4.9
+2°C column initial oven temperature	5.1
